# “It’s all about trust”: reflections of researchers on the complexity and controversy surrounding biobanking in South Africa

**DOI:** 10.1186/s12910-016-0140-2

**Published:** 2016-10-10

**Authors:** Keymanthri Moodley, Shenuka Singh

**Affiliations:** Centre for Medical Ethics and Law, Department of Medicine, Faculty of Health Sciences, Stellenbosch University, Tygerberg, South Africa

**Keywords:** Biobanking, Researchers, South Africa, Trust

## Abstract

**Background:**

Biobanks are precariously situated at the intersection of science, genetics, genomics, society, ethics, the law and politics. This multi-disciplinarity has given rise to a new discourse in health research involving diverse stakeholders. Each stakeholder is embedded in a unique context and articulates his/her biobanking activities differently. To researchers, biobanks carry enormous transformative potential in terms of advancing scientific discovery and knowledge. However, in the context of power asymmetries in Africa and a distrust in science born out of historical exploitation, researchers must balance the scientific imperative of collecting, storing and sharing high quality biological samples with obligations to donors/participants, communities, international collaborators, regulatory and ethics authorities. To date, researcher perspectives on biobanking in South Africa have not been explored and documented.

**Methods:**

In-depth qualitative interviews were conducted with a purposive sample of 21 researchers – 8 in the Western Cape, 3 in Gauteng and 10 in Kwa-Zulu Natal. Interviews lasted approximately 40–60 min and were audiotaped with consent. Thematic analysis of the transcribed interviews was conducted by the co-authors.

**Results:**

Researchers articulated serious concerns over standardised regulatory approaches that failed to consider the heterogeneity of biobanks. Given that biobanks differ considerably, guidelines and RECs need to stratify risk accordingly and governance processes and structures must be flexible. While RECs were regarded as an important component of the governance structure researchers expressed concern about their expertise in biobanking. Operational management of biobanks was regarded as an ethical imperative and a pre-requisite to building trust during consent processes. While broad general consent was preferred, tiered consent was thought to be more consistent with respect for autonomy and building trust. Material Transfer Agreements (MTAs) were often lacking when biosamples were exported and this was perceived to impact negatively on trust. On the other hand, researchers believed that authentic community engagement would help to build trust.

**Conclusion:**

Building trust will best be achieved via a system of governance structures and processes that precede the establishment of a biobank and monitor progress from the point of sample collection through to future use, including export. Such governance structures must be robust and must include comprehensive national legislation, policy and contextualised guidelines. Currently such governance infrastructure appears to be lacking in many African countries including South Africa. Capacity development of all stakeholders including REC members will enhance expeditious and efficient review of biobanking protocols which in turn will reinforce trust in the researcher-donor relationship. Science translation and community engagement in biobanking is integral to the success of biobanking in South Africa.

**Electronic supplementary material:**

The online version of this article (doi:10.1186/s12910-016-0140-2) contains supplementary material, which is available to authorized users.

## Background

Biobanks could be described as a “discursive practice that is vague and open to articulation because each particular stakeholder embedded in a particular context articulates its biobanking activities differently” [1]. Furthermore, biobanks are located at the intersection of science, genetics, genomics, society, ethics, the law and politics. This multi-disciplinarity has given rise to a new discourse in health research involving diverse stakeholders.

African genetic diversity lies at the core of the controversy that surrounds data and sample mining. Our samples are highly sought after internationally and the unidirectional flow of samples out of Africa has raised huge concerns around exploitation of vulnerable communities and countries. In an attempt to stem the tide of sample exportation the Human Health and Heredity Africa (H3 Africa) project funded jointly by the National Institutes of Health (NIH) and the Wellcome Trust, seeks to develop scientific capacity in Africa by encouraging African scientists to develop biorepositories in various African countries including South Africa. If successful, this venture will present an unparalleled opportunity for researchers and healthcare in Africa.

Biobanking has become a core resource for medical researchers as it has enormous transformative potential. However, researchers must also be mindful of the intricate web of ethical and social complexities inherent in the collection, storage and future use of biospecimens, especially in Africa, given the power asymmetries at play in international collaborative research relationships and a historical context of exploitation. Public opinion is cautious and trust in medical research in South Africa is waning. This crisis in trust has been fuelled by a history of exploitation in medical research that exploits vulnerabilities of developing world communities. It is also fuelled by the deep cultural significance that South African communities attach to blood and other biological samples [2].

Although most literature on ethical concerns related to biobanking focuses on consent [2–6], other concerns such as governance, community engagement, international collaboration and sample sharing are relevant [6–10]. Furthermore, several stakeholders are central to biobanking and eliciting their multiple and diverse perspectives is important. Previous studies on biobanking have explored participant [2, 10], REC member [11], patient [12] and media perspectives [13]. Some papers have explored the views of researchers who collect and use samples in resource rich countries [14–20]. To date there has been no published empirical work on researcher perspectives on biobanking in Africa. Consequently, this qualitative study was undertaken to explore perspectives of researchers working with biospecimens and/or biobanks in South Africa.

## Methods

In-depth qualitative interviews were conducted with 21 researchers – 8 in the Western Cape, 3 in Gauteng and 10 in Kwa-Zulu Natal. Most researchers (12) were male and 9 were female. Respondents were medical and scientific researchers, biobanking experts and governance experts working across different disciplines: virology, haematology, immunology, pathology, HIV and Tuberculosis. Purposive sampling was used to identify researchers for the individual face-to-face in-depth interviews. An interview schedule was designed to elicit participants’ understanding and opinions of ethical considerations in the use of biological material for research purposes and biobanking in particular (Additional file 1). The guide was based on existing literature [1–10, 15–19].

For the recruitment strategy, all identified researchers were contacted via email, as an introduction to the proposed study. The principal investigators thereafter set up face-to-face interviews based on the participants’ availability and willingness to participate in the study (Additional file 1). The consent process was conducted verbally and in writing. Interviews were digitally recorded and lasted an average of 30–45 min. The interview schedule comprised open-ended questions that focused on the following broad areas: scientific and ethical concerns about biobanking; governance; informed consent; data and sample management, export and sharing. Interviewees were also given the opportunity to make recommendations for the use of biological material in South Africa and to provide policy inputs for the National Health Act (No. 61 of 2003). During the data analysis process, audio-recorded interviews were first transcribed verbatim and a data clean-up process was applied. The narrative from each interview transcript was subjected to thematic analysis [21]. Both interviewers (KM and SS) read through the transcripts to extract themes (analyst triangulation). The data were analysed by the two authors independently and then integrated via discussion.

## Results

Seven main themes emerged from the data. The competence of RECs in reviewing biobanking protocols was a common concern. A careful analysis of the risks and benefits of biobanking was regarded as a prerequisite to considering other ethical issues. Amongst these, consent emerged as a contentious and unresolved issue. Governance was regarded as a multi-layered concept with national guidance and regulations often poorly developed. Likewise, the absence of Material Transfer Agreements made export of samples challenging even on the African continent. Most participants agreed that sound operational management and sustainability of biobanks was an ethical imperative. Finally, community engagement was regarded as a critical process to build trust. Each of these themes is discussed in detail.

### Research ethics committee competence

As a point of departure, researchers interviewed in this study all referred to the heterogeneity that exists in the field. They explained that biobanks may range from small collections of disease specific blood and tissue samples to large scale general population based collections of blood exceeding a million samples.

Different environments maintain specific biobanks… “study specific biobanks so it’s not a biorepository in the true sense of the word”.

“Virology keeps biobanks for 3 reasons: for diagnostic purposes, to validate tests and to introduce or evaluate a new diagnostic method with samples such as for Viral Load monitoring. However if we later want to do resistance testing, consent has not been obtained. We can get a waiver of consent via the REC for anonymised samples but if we anonymise we lose value to individual patients – the REC may not understand this”.

Others commented that RECs must be able to understand heterogeneity of biobanks and stratify the review according to the risks related to volume and type of specimens handled. Many researchers felt that RECs needed to take heterogeneity of biobanks into account in decision-making and governance. There were also concerns about careful risk-benefit calculations in REC deliberations.

### Risk-Benefit ratios in biobanking

Researchers were sensitised to the risk-benefit calculations that precede regulatory decision-making in biobanking. They distinguished between individual benefit and public health benefit. Most of the respondents were extremely confident about the scientific and clinical benefits inherent in biobanking. With HIV viral load samples one can later look for resistance, neutralising antibodies or mutations associated with antiretroviral treatment failure. However, this potential individual benefit for the patient is lost if samples are anonymised obviating the need for re-consenting participants for future use of samples. On the other hand, with some diseases, public health benefits are great, hence an opt-out system of consent or waiver of consent would be justified. A distinction was also drawn between short term and long-term benefits. Prospectively biobanking high quality samples with associated data in a retrievable manner will have an enormous impact on streamlining and accelerating medical scientific research in the future:

“We are banking for the future…the benefit in all this is for the next generation.”

“I don’t know what the research question is going to be therefore I must bank in such a way that I can ask any research question in the future.”

A number of risks were identified. This included the concept of over-researched communities:

“There is competition for participants by different research groups…there are actually not enough TB [Tuberculosis] patients in Cape Town for the number of research projects. It’s over researched. People are fighting over TB patients. There is competition between basic science and clinical trial research. Some patients are selling sputum outside the clinic, so that negative patients can enrol to get a box of biscuits or a grocery voucher”.

Concern was expressed over the prospect of commercialisation which was regarded as a significant risk. In particular, respondents argued that selling samples to a pharmaceutical company should not be allowed:

“Human tissue is this untouchable Holy Grail that you can’t mess around with, you cannot abuse it, you cannot sell it, and you cannot make a profit out of it.”

Infectious disease specimens were regarded as a risk to biobank staff and patients alike. The need for robust infection control measures in biobanks was stressed.

An important benefit reported in the field of infectious disease was that super- spreaders of HIV could be identified….sequence data could reveal the patient even if the sample were to be anonymised. Researchers therefore referred to the “myth of anonymity”. While this researcher mentioned the identification of super-spreaders as a benefit, this benefit referred to preventing further spread of HIV in an attempt to control the epidemic. Deliberate transmission of HIV has not been criminalised in a specific law in South Africa. However, biobanked specimens could potentially be used in a legal investigation, if required by the courts.

Finally, stigma associated with genetics and genomics research was regarded as a specific risk to patients making genetic and genomic biobanking more challenging. Concerns around such risks and benefits would need to be included in consent processes.

### Preferred consent models

Discussions around consent ranged from broad consent to tiered consent with one researcher referring to dynamic consent. Most researchers indicated a preference for one time broad consent that would allow future use of samples. Re-consenting participants was regarded as impractical and resource intensive:

[We have to] “balance the time and effort spent on consenting versus time spent on research”

“It would cost millions to re-consent participants…is it more unethical to keep samples or destroy them?”

“Reasonable efforts are made to contact patients… but in practice this is difficult because mobile telephone numbers change and patients relocate frequently [in South Africa].”

Some researchers/scientists who work closely with clinicians expressed concern about the sustainability of detailed consent processes:

“Clinicians feel they have the trust of patients and would like to obtain consent – whether that’s a sustainable option remains to be seen. The consent form is rather comprehensive and it will take time. While clinicians may initially speak to patients to get their agreement, formal consent can be obtained by a nurse, scientist or technologist from the biobank who is trained to do the consent”.

On the other hand, researchers working with indigenous populations in Southern Africa were quite confident that some communities would not subscribe to broad consent:

“…the San Council would not give broad consent, they would only give fairly narrow specific consent but they would not be averse to re-consenting if there was something worthwhile to be done”.

A tiered consent model which included specific consent and broad consent would be applicable in such cases and would increase trust as choices are provided to participants.

Citing technological advances as a means to improve consent processes, one researcher explained how

“we are moving to the era of dynamic consent and dynamic participation…with the age of technology…that’s possible. You will receive constant notifications on your cell phone that your samples have now been created into cell lines or they have been submitted to a pharmaceutical company who are now creating a new drug.”

Moving away from models of consent, some researchers expressed concern about the actual information contained in consent forms:

“Consent forms should evolve in consultation with Community Advisory Boards so that the consent form can actually contain the information that would be really important from the patient and community perspective.”

“At the moment the consent form is the product of the researcher/research team and biobank perspectives… it contains literature from an ethics and legal perspective…it may not be important from the patient’s perspective.”

While many researchers believed that anonymization of samples could impede return of individual results to participants others referred to the “myth” of anonymization:

“Withdrawal of samples and return of results, even if samples were anonymised, would be possible using genetic signatures. H3Africa is working on a SNP profile for African ethnic groups.”

### Multiple levels of governance is imperative

Collectively, our respondents contributed different aspects of governance to the discussion around biobanking.

“Governance structures are multi-tiered…international bodies like WHO and United Nations, WMA…Helsinki. Then you have societies like ISBER and ESBB and high level governance structures with guidelines and policies. On the continent, the African Union and SADC region policies. Nationally, South African policy…Department of Health…University policies…a microenvironment of board members and trustees and community engagement”.

Some respondents were critical of the existing legislation in South Africa with respect to biological samples:

“The lack of regulatory standards is a problem and legislation has not really been quite clear on biobanking research”. [The] “definition of tissue is problematic and excludes plasma which has no cells so is excluded from the legislation but plasma may have viruses”.

The tissue banking legislation has been written for therapeutic biobanks – bone banks, stem cell banks, heart valves, gametes, corneas…. these are different from research biobanks.

“Biobanks and tissue banks are not the same thing. We need a separate set of regulations for biobanking which could fall under chapter 8 of the National Health Act”.

Although the REC was seen as a governance structure by most researchers, some commented that biobanking protocols are

“evaluated by people who are not experts within the field and do not know the implications of what we are doing….people who do not differentiate between viral genomics and human genomics…we need expert panels that have expertise to review biobanking protocols”.

Finally, some researchers contended that we need to conduct an audit at a national level:

“…we need to go around and find out where the biobanks are, how have samples been stored, make sure ethics approvals are appropriate, is there a global unrestricted use of samples….we will have to look at the governance structures.”

In this respect there was considerable discussion around governance involved in the export of samples.

### Export and Material Transfer Agreements (MTAs)

Discussions around export of samples and MTAs elicited strong opinions. Researchers insisted that patients must be informed that their samples will be sent to the United States (US) and/or Europe with contracts, MTAs and export permits. A significant concern related to the lack of a national MTA in South Africa.

“MTAs are drawn up by the legal department. The samples cannot be handed over to anybody but there’s no way I can monitor what they do with the samples. We send it based on trust. Some researchers in Europe and the US try to bully us….they think all African institutions are backward and they are not very respectful towards the sample providers. They are quite taken aback when we actually insist on an MTA.”

Preventing unilateral drift of samples out of Africa and retaining intellectual property rights on the continent was generally regarded as important. With respect to the H3Africa project and the African Malignancy Consortium (AMC), the “biobank will provide specimens to researchers from anywhere in the world with an approved research protocol….but the Principal Investigator or co-PI must be from the African continent”.

However, even moving samples within the continent is challenging as some African countries have MTAs while others do not. Legislation with respect to export differs from one country to the next making cross-border transfer of samples almost impossible. This was regarded as important enough to erode trust in biobanking.

### Operational management and sustainability of biobanks as ethical imperatives

Infrastructure and security needs are considerable for biobanking. Researchers highlighted the critical need for electricity and back-up power supplies. If a biobank collection of samples is housed in two different buildings, two generators are required. In South Africa in recent times

“with power interruptions and load-shedding the generator came on but did not deliver power and that led to a huge crisis. Five of the ten freezers broke down, each freezer costs R120 000 (about US $10 000)….you can recover reagents but not samples and samples cannot be insured. So we got a 24 h surveillance system to monitor the temperature and a whole team that is available 24 h a day, every day of the year, to monitor our freezers”. Operational costs are high “it costs R1.25 per sample per month to store a sample in a -80° Celsius freezer and double that (R2.50) to store in liquid nitrogen”.

Retrieval systems must be optimised. Lost samples were regarded as a violation of promises made to participants during consent processes.

“You’ve got to have buy in from your participants… and colleagues because biobanking is very expensive. If you are going to make biobanking worthwhile then you must introduce the concept of centralised banking in institutions or regions”.

Sustainability of a biobank requires stable and continuous income streams and reliable funding sources. Respondents expressed strong concerns that lack of robust operational management of biobanks would result in wasted samples and abandoned, liquidated or sequestrated biobanks in financial distress. This would most certainly undermine donor trust. Integrity and quality of samples, good business practice and governance were regarded as non-negotiable ethical requirements for biobanking.

### Community engagement as a research priority to build trust

Researchers were unanimous about the value they placed on community engagement as a pre-cursor to building trust.

“We have to take participants along with us. That involves an extensive amount of engagement involving them in empirical research: what are their views and opinions? What are their cultural issues around biobanking and the cultural issues around the use of tissue and the movement of tissue to other countries and into the commercial space?”

Generally, researchers emphasized the meaning that cells, blood, tissues and organs held culturally and this had to be explored with participants and patients:

“If we are going to create cell lines, you know the whole African concept about your spirit lives in your cells, your cells are still alive and when I die I want my cell lines to be brought back. Once your cell lines have gone, they are gone. You can’t ever bring them back.”

In particular, a few of the respondents in this study shared their experiences in working with indigenous communities such as the San communities in Southern Africa:

“With the San community, researchers must first meet with community leaders, get to know them, build trust and then, in the presence of community leaders explain to the community what it is that one is aiming to do and then get the community to vote…once the community agrees then you have to explain to individual participants and get individual consent. After the study you need to go back to the community, first to community leaders to explain the implications of your findings, then explain to the community”.

Benefit sharing was expressed as follows: “if one could contribute to the education of the San community by building a school or providing bursaries to send people to university then I think that is fine. That’s a form of benefit sharing.”

Various suggestions on how to engage with communities were proposed - community newspapers, educational videos, strong advocacy groups that would “ensure good representation and trust”. You have to engage in widespread community activity whereby the community knows what you are doing and are reassured about the integrity and trust, “because it’s all about trust”. In addition, annual feedback to the community was regarded as essential.

Concern was expressed that funding of community engagement is still too low and that researchers and funders need to include this as a separate budget item in grant applications.

## Discussion

Some of the concerns raised in this study of researchers echo findings in other studies from resource rich environments. However, many concerns deviate significantly.

The most striking sub-theme underlying the major themes that emerged in this study was the importance of building trust with communities. In some countries biobanks are being established in a “context of heightened concerns” about a “decline in trust” in scientists, authorities and experts in the regulatory systems governing biotechnology innovations [22]. Recently the tension has been exacerbated by participants exercising individual and group autonomy and demanding return of their blood specimens [23]. Several authors cite trust as a central component of the research participant-researcher relationship [2, 24–26]. In developing countries, failure to engage with communities has been identified as a reason for erosion of trust in researchers [27]. Similarly, Barchi et al., in their study on REC members in Botswana, identified lack of trust as a significant challenge and also cite failure to engage with communities as a cause of the erosion of trust [11]. Dilution of trust in the researcher – donor relationship may occur when samples are exported for unknown future use in foreign countries [28]. Respondents in this study concurred that trust underscores all biobanking endeavours and is critically predictive of success.

Governance of biobanks will require understanding of the “immense diversity found in organizational features” [29]. Considerable heterogeneity exists in the field and biobanks may range from small collections of disease specific blood and tissue samples to large scale general population based collections of blood and other tissue. Respondents in this study identified the dissonance between the type of biobank, the nature of proposed research and the ability of REC members to appreciate the risk. This was perceived as a major obstacle to obtaining approval.

A standardized approach in reviewing all protocols involving biobanks in the same way has the potential to lead to generalization of risks and over-regulation of research. They argued that it is important for RECs to be able to stratify risk according to type of biobank being proposed or used. Small, local, non-communicable disease specific biobanks (such as exist for hypertension or diabetes) within institutions tend to use stored samples for specific research limited to that disease area in the future. If patients have consented to such specific future use, such research carries lower risk than a project involving the collection of large volumes of specimens from healthy participants for undefined broad future use including export to other countries.

Similarly, specimens collected in the context of highly infectious disease outbreaks such as Ebola carry higher risk in terms of risk to biobank personnel, transportation, export and security risks. Often small collections of samples are over-regulated while large-scale biorepositories and sample collections that are potentially infectious are under-regulated. Thousands of specimens left West Africa during the recent Ebola outbreak and the fate of these specimens is still unclear and unregulated [30].

Many respondents in this study expressed frustration about the decisions made by RECs based on a lack of understanding of biobanks and the potential future use of biospecimens in research. Similar frustration was expressed by researchers in the United Kingdom who participated in 4 focus group discussions (FGDs) in 2011. In fact, in that study, the challenges experienced by researchers with regulatory bodies was the most striking finding that emerged from the FGDs. These challenges were exacerbated in international studies where sharing of samples and data across borders was involved. In such studies the REC approval process was considerably lengthened [16]. Such concerns have been raised in previous publications examining governance in Africa [8, 9]. Likewise, in our study, the lack of a national MTA in South Africa and in other African countries was regarded as holding the potential to seriously delay biobanking and biobanking research on the continent and to undermine trust in international studies.

At a national level in South Africa, governance was cited as an obstacle due to regulations that are not sufficiently comprehensive and even absent in the arena of biobanking for research. This concern echoes findings in previous publications [8, 9]. The South African research ethics regulatory structure comprising a National Health Research Ethics Council (NHREC) with an oversight and accreditation function over the 33 RECs registered with it, requires that the national body establish guidance on biobanking review and capacity development of REC members in this field. It is also the obligation of the NHREC to strongly motivate, via the Department of Health, for the development of legislation on biobanking in South Africa. Likewise, selection of REC members in South Africa must be reviewed with attention devoted to expertise, research experience and commitment to research ethics review. Training opportunities for REC members have been repeatedly emphasized yet this often remains a neglected issue at an institutional level.

Given the low levels of science literacy in South Africa and the challenges posed in consent processes for a wide range of research, consent for biobanking was prominently discussed by respondents in this study. In keeping with published studies on researcher perspectives which reflect a preference for broad consent with open-ended future use [19], the respondents in this study strongly supported broad consent. This finding was echoed by Whitley et al. where broad consent was preferred due to the challenges posed by predicting future research use [16]. However, many respondents reflected a deep concern for the principle of respect for persons by suggesting flexibility in consent options, engaging with communities and respecting cultural contexts and norms, especially of indigenous populations. There was a sensitivity to previous exploitation of research participants in Southern Africa and the consequent enforcement of rules in specific indigenous communities as a protective measure. The approach of community consultation for research required by the San Council in Southern Africa is unique in the context of research ethics review systems in the country that are based on a more individualized approach to personhood. It is however important to note that community consent will not replace individual consent. The San Council views the two levels of the consent process as complementary.

Few previous studies have explored dynamic consent where participants are able to liase with a biobank on a long-term basis using a mobile application and give input on future use of their donated biospecimens [16]. However, researchers proposed this as a strategy to build trust in South Africa given our large mobile phone network and the possibility of using mobile applications to engage biobank donors in discussions around future use of donated biospecimens. It could be argued that dynamic consent carries the potential to erode privacy. However, engaging with biobank donors in this manner will involve the creation of secure platforms to ensure confidentiality to the extent possible and privacy will also respected. Donors will be contacted discretely and only when absolutely necessary. Despite the convenience of broad consent to researchers and biobanking custodians, a significant proportion of research participants surveyed in South Africa have articulated a desire to be consulted on future use of their samples [10]. Tiered consent affords research participants the opportunity to make this choice while dynamic consent allows for implementation of this choice.

This expressed need to involve biobank donors in decision-making could be a response to the claim that the traditional science-society relationship premised upon a “clear separation between expert and lay knowledge” has been exposed in biobanking [22]. Respondents in this study were acutely aware of the need to bridge this gap by engaging with communities to facilitate consent processes in biobanking. Various suggestions were made to ensure science translation and some of these community engagement tools have been described elsewhere [31]. However, involving patients as active partners in biobanking, deliberative democracy activities with communities [31] and patient led biobanks [12] were not mentioned. Even though such measures have been implemented in the United States and Europe, the authors caution that it was not without challenges including financial and human resources [12, 30]. Implementing such a “cultural revolution in health research” [12] in South Africa is to be encouraged. However, significant education of educationally disadvantaged communities is a precursor to this intense level of public engagement. It is unclear if new models of public engagement will change the asymmetrical power relations between researchers/scientists and civil society [22] in South Africa.

Finally an important and repeated comment in this study was the “myth of anonymization”. While RECs viewed anonymization as protective of participant rights to privacy, technological advances in genetics and genomics may make anonymization impossible. Biobanking is widely cited as having the potential to create ethical concerns. Privacy is a prominent concern voiced by donors. It is imperative that the question of coding and anonymization be addressed so that consent processes are not perceived as misleading and hence a barrier to the development of mutual trust between researchers/scientists and civil society.

Although this is the first study in South Africa to reflect the perspectives of researchers, it has surveyed respondents in 3 regions only. Future research that casts the net more widely in a geographic sense has the potential to increase generalisability of the study. Surveys in other African countries will also yield interesting comparative data. Given that this is the first survey of researchers in South Africa, a wide range of concerns was raised in this study. Future research could narrow the focus to examine some of these issues in greater depth. In particular, benefit sharing was briefly mentioned with respect to indigenous communities. However this represents a study in its own right and could potentially be explored especially in resource poor settings. Likewise the myth of anonymity should be further explored.

## Conclusion

Given the history of exploitation in South Africa as a result of apartheid and colonialism and given the general lack of trust of scientists, building trust will best be achieved via a system of independent governance structures and processes that precede the establishment of a biobank and monitor progress from the point of sample collection through to future use, including export. Such governance structures must be robust and must include national legislation, policy and contextualised guidelines. Currently such governance infrastructure appears to be lacking in many African countries including South Africa. Capacity development of all stakeholders including REC members will enhance expeditious and efficient review of biobanking protocols which in turn will reinforce trust in the researcher-donor relationship. All efforts to improve trust will lead to enhanced legitimacy of biobanking in South Africa. Establishing relationships with international collaborators based on trust, equity and integrity is also critical for sharing of samples and data.

## Additional file

Additional file 1: Figure S1. Interview Guide. (DOCX 13 kb)

## Abbreviations

AMC: African Malignancy Consortium
ESBB: European, Middle Eastern and African Society for Bio-preservation and Biobanking
H3 Africa: Human Health and Heredity Africa
HIV: Human immunodeficiency virus
ISBER: International Society for Biological and Environmental Repositories
MTA: Material transfer agreement
NHREC: National Health Research Ethics Council
NIH: National Institutes of Health
REC: Research Ethics Committee
SADC: Southern African Development Communities
SNP: Single nucleotide polymorphism
TB: Tuberculosis
US: United States
WHO: World Health Organisation
WMA: World Medical Association
