# Supplementary material for: “It’s all about trust”: reflections of researchers on the complexity and controversy surrounding biobanking in South Africa
Source: BMC Med Ethics. 2016 Oct 10;17:57. doi: 10.1186/s12910-016-0140-2 (PMC5057490; doi:10.1186/s12910-016-0140-2)
Supplement: Additional file 1: Figure S1. — Interview Guide. (DOCX 13 kb) [file 12910_2016_140_MOESM1_ESM.docx]

**ADDITIONAL FILE 1**

**FIGURE 1**

**INTERVIEW GUIDE : RESEARCHERS**

**Demographics:**

1. Researcher/ Pathologist
2. Discipline______________________________________________________
3. Number of years working in your field________________________________
4. Private sector/Public sector________________________________________

**Specific Questions:**

1. How are you involved in obtaining or processing biological samples?
2. What scientific concerns do you have with respect to collecting/storing biological samples?
3. What ethical concerns do you have about collecting/storing biological samples?
4. What type of consent do you think should be obtained from participants/patients?
5. Please justify your suggested format of consent?
6. How do you feel about storage of samples in a biobank?
7. How do feel about secondary use of specimens for research?
8. Do you have any concerns about exportation of samples?

Yes/No and probe reasons

1. What suggestions do you have for the NHREC in terms of use of biological samples in South Africa?
2. What suggestions do you have for chapter 8 of the National Health Act?
3. Do you have any other comments or questions?
